# Sport Concussion Assessment Tool-5th Edition (SCAT5): Normative Reference Values for the National Rugby League Women's Premiership

**DOI:** 10.3389/fspor.2021.653743

**Published:** 2021-05-26

**Authors:** Grant L. Iverson, David R. Howell, Ryan Van Patten, Paul Bloomfield, Andrew J. Gardner

**Affiliations:** ^1^Department of Physical Medicine and Rehabilitation, Harvard Medical School, Boston, MA, United States; ^2^Department of Physical Medicine and Rehabilitation, Spaulding Rehabilitation Hospital, Charlestown, MA, United States; ^3^Spaulding Research Institute, Charlestown, MA, United States; ^4^Sports Concussion Program, MassGeneral Hospital for Children, Boston, MA, United States; ^5^Home Base, A Red Sox Foundation and Massachusetts General Hospital Program, Charlestown, MA, United States; ^6^Sports Medicine Center, Children's Hospital Colorado, Aurora, CO, United States; ^7^Department of Orthopedics, University of Colorado School of Medicine, Aurora, CO, United States; ^8^National Rugby League, Moore Park, NSW, Australia; ^9^Hunter New England Local Health District Sports Concussion Program, Newcastle, NSW, Australia; ^10^Centre for Stroke and Brain Injury, Calvary Mater Hospital, School of Medicine and Public Health, University of Newcastle, Waratah, NSW, Australia

**Keywords:** rugby league, brain concussion, head injuries, baseline survey, clinical assessment, women athletes

## Abstract

**Objective:** To establish normative reference values for the Sport Concussion Assessment Tool-5th Edition (SCAT5) for the new National Rugby League Women's Premiership.

**Methods:** Preseason SCAT5 baseline testing was administered individually to all National Rugby League Women's Premiership players (*N* = 117). Testing was completed by the medical staff. Normative reference values were calculated for the components of the SCAT5, including the Standardized Assessment of Concussion, modified Balance Error Scoring System, and the Symptom Scale. A small case series of players who sustained concussions were included to illustrate the use of the new normative data.

**Results:** The median Standardized Assessment of Concussion total score was 27.0 (M = 26.9, SD = 2.1). The median modified Balance Error Scoring System score was 2.0 (M = 2.4, SD = 2.2). The median number of symptoms score was 1.0 (M = 3.2, SD = 4.7) and the median symptom severity score was 2.0 (M = 5.4, SD = 8.2). The most common baseline symptom was fatigue or low energy (33%), followed by trouble sleeping (24%), headache (23%), neck pain (22%), and difficulty remembering (21%). In the total sample, 41% reported no symptoms. The clinical interpretation of these new normative data to a case series of women with concussions is provided.

**Conclusions:** Normative reference values are provided for the SCAT5 for women who are professional rugby league players. Using these normative data will improve clinical interpretation of SCAT5 scores following a concussion.

## Introduction

Rugby League is a fast paced full contact collision sport (Gabbett, 2005) with frequent tackling (King et al., 2010) and a relatively high rate of concussions (Gardner et al., 2014). Although distinct, the game is similar to Rugby Union—which is played in many countries. The National Rugby League (NRL) is the elite professional level club rugby league competition in Australia. A new professional league for women in this sport had its first season in 2018. The league has a concussion management protocol that includes baseline, preseason medical evaluations of all players using the Sport Concussion Assessment Tool-Fifth Edition (SCAT5) (Echemendia et al., 2017) and then follow-up testing with the SCAT5 if a player is injured during the season.

The SCAT5 (Echemendia et al., 2017) has been promoted by the Concussion in Sport Group as a standardized acute clinical assessment for athletes suspected of concussion (McCrory, 2005; McCrory et al., 2009, 2013). The SCAT5 includes an on-field and off-field evaluation (see Table 1). The standardized off-field tests are used to measure an athlete's subjectively experienced symptoms, cognitive functioning, and statistic balance and postural stability. The SCAT5 is comprised of existing tests such as the Standardized Assessment of Concussion (SAC) (McCrea et al., 1998), modified Balance Error Scoring System (mBESS) (Riemann and Guskiewicz, 2000; Buckley et al., 2018), and a modification of the Post-Concussion Symptom Scale (Guskiewicz et al., 2013; Echemendia et al., 2017). The SCAT has been, and is, widely used in professional and amateur sports (Yengo-Kahn et al., 2016; Cochrane et al., 2017; Fuller and Raftery, 2018; Katz et al., 2018; Fuller et al., 2020). It is used to document the acute effects of concussion and to monitor recovery from the injury (Echemendia et al., 2017).

**Table 1:** Components of the SCAT5 (Echemendia et al., 2017).

**Immediate or on-field assessment**	
Step 1: Red Flags	Observable signs indicating the need for immediate and safe removal from participation and medical evaluation (e.g., loss of consciousness, seizure, deteriorating conscious state, vomiting)
Step 2: Observable Signs	Observed signs of possible concussion, such as lying motionless, motor incoordination, and disorientation or confusion
Step 3: Memory Assessment Maddocks Questions	Five sport-specific orientation and amnesia questions (e.g., who scored last in this match?)
Step 4: Glasgow Coma Scale (GCS) and Cervical Spine Assessment	The GCS is used in rare circumstances to document neurological deterioration and more severe injury; the cervical assessment documents neck range of motion, limb strength, and limb sensation
**Office or off-field assessment**	
Step 1. Athlete Background	Demographic variables, self-reported concussion history, developmental and health history, and medications
Step 2. Symptom Evaluation	22 symptoms on a dimensional scale from 0 (none) to 6 (severe)
Step 3. Cognitive Screening (Standardized Assessment of Concussion)	Cognitive assessment of time orientation, immediate memory, concentration, and delayed recall
Step 4. Neurological Screen and Balance Examination (Modified Balance Error Scoring System; mBESS)	Cervical/neck examination, eye movements, finger-to-nose test, and tandem gait, followed by static balance and postural stability testing with the mBESS

*For baseline preseason testing, the scores that were derived for the present study were the symptom scores, cognitive screening scores, and the mBESS scores*.

There are two distinct ways to interpret SCAT5 performance following a suspected or known concussion. First, the clinician can compare post-injury scores with age-, sex-, and sport-specific normative data (Fuller and Raftery, 2018; Katz et al., 2018; O'Connor et al., 2018; Petit et al., 2020). Second, the clinician can compare post-injury test scores to the individual's personal pre-injury baseline data. The clinician might also choose to use both methods when possible, in order to allow for interpretation of the athlete's scores from both a relative (i.e., within-person) and normative (i.e., between-person) standpoint—and to increase confidence in one's clinical interpretation of the results. Therefore, in this context, the purpose of this study is to provide normative SCAT5 reference values for women who are professional rugby league players.

## Methods

### Participants

All 117 women registered in the National Rugby League Women (NRLW) from the 2018 and 2019 seasons were included in this study. For those women completing the SCAT5 twice (i.e., each year), only the results from their first time taking the test were included in this study. The NRLW was established in 2018 and has four teams, with a total of seven games played per season. Team doctors administered the SCAT5 prior to the first game of the season. Demographic information was not recorded as part of the baseline preseason testing. To estimate age, we identified the ages of all women by reviewing individual player profiles on the NRL website (https://www.nrl.com/players/?competition=161). Their mean age, as of January 1, 2019, was 25.9 years (SD = 5.5, interquartile range = 22–29, range = 17–42). Testing was conducted prior to the beginning of the season, by the medical staff, and the athletes were healthy at the time of testing (no athlete, for example, was recovering from a concussion at the time of preseason testing).

### Measure

The SCAT5 includes a series of individual components and tests that are designed to evaluate an athlete who is suspected of or known to have sustained a concussion. The components of the SCAT5 on-field and off-field assessment are summarized in Table 1. When used for baseline preseason testing, the athlete completes the symptom ratings based on “how she typically feels.” The concussion symptom evaluation consists of 22 symptoms that are graded on a dimensional scale from 0 (none) to 6 (severe). The cognitive assessment is based on the SAC (McCrea et al., 1998), which assesses time orientation, immediate memory (ability to learn a list of either 5 or 10 words, over 3 trials), concentration (digit span backwards), and delayed recall (for the word list). Baseline testing also includes a measure of static balance and postural stability using the modified version of the mBESS (Riemann and Guskiewicz, 2000). The third edition of the SCAT, the SCAT3, included four trials of the tandem gait test. That component was retained by the women's league as part of their baseline preseason testing program and those normative scores are provided for the current study. In the future, these normative data will be expanded and updated to reflect the league transition to the 10-item word list and using an untimed, pass-fail tandem gait test.

### Procedure

SCAT5 preseason baseline testing is mandatory for all registered NRLW players in order to obtain individual baseline performance on this measure. The testing was conducted by the team doctor. If any score on a subcomponent of the SCAT5 was incomplete, the data for that participant were excluded from the analyses of that subcomponent.

### Statistical Analyses

Data were analyzed with Stata version 15 (StataCorp, College Station, TX). SCAT5 descriptive statistics, calculated for each component, include the mean (M), median (Md), standard deviation (SD), and interquartile range (IQR). Normality of each of the SCAT5 components was assessed with histograms and using Shapiro-Wilks tests. Normative classification ranges were determined for each component, with cutoff values selected to be similar to the Wechsler model (Wechsler, 2008), a commonly accepted convention in neuropsychological assessment. The Broadly Normal range was defined as the 25–74th percentiles (the IQR), the Below/Above Average range was defined as the 10–24th and 75–90th percentiles, the Unusually Low/High range was defined as the 3rd−9th and 91st−98th percentiles, and the Extremely Low/High range was defined as < 3rd and >98th percentiles. Additionally, the directionality of several of the individual components differ and this is reflected in the presentation of results. That is, higher scores on some variables reflect greater symptoms (e.g., symptom severity), greater error rates (e.g., mBESS total errors), or worse test performance (e.g., Tandem gait: best time), and, consequently, worse overall functioning. On the other hand, higher scores on the SAC subtests all reflect better cognitive performance, and better overall functioning.

The proportion of the sample endorsing each of the 22 SCAT5 symptoms was calculated and stratified by symptom severity (none, mild, moderate, severe). A cumulative frequency distribution was generated to reflect the number of participants who endorsed symptoms, 0–20+. We included pro forma psychometric analyses examining the intercorrelations between different SCAT5 test scores. Spearman (non-parametric) correlations were conducted to examine the associations between the following SCAT5 components: (i) symptom total and symptom severity, and (ii) symptom severity, SAC total score, mBESS total errors, and tandem gait: mean time.

### Ethical Considerations

The research program has previously been endorsed by the Rugby League Research Committee. This study was approved by the Institutional Human Ethics Committee (Ref No. H-2012-0344).

## Results

### Baseline SCAT5 Scores

Baseline results for the SCAT5 components are presented in Table 2. All SCAT5 components were non-normally distributed, based on both visual inspection of histograms and Shapiro-Wilks tests (*p*s < 0.03). The individual cutoff values and associated normative classification ranges for the SCAT5 components are shown in Table 3. On the SAC delayed recall component, 8% (n = 9) of the sample obtained a perfect score. Digits backward was perfectly completed by 38% (*n* = 45) of participants. The months in reverse were stated correctly by 85% (*n* = 100) of the sample.

**Table 2:** Summary of baseline results for women professional rugby league players for each SCAT5 test component.

**Test component**	**Scale**	***n***	**Mean**	**SD**	**Median**	**IQR**	**Range**
Symptom severity score	0–132	117	5.4	8.2	2	0–8	0–45
Total number of symptoms	0–22	117	3.2	4.7	1	0–4	0–20
SAC total score	0–30	117	26.9	2.1	27	26–29	21–30
Orientation	0–5	117	4.8	0.4	5	4–5	3–5
Immediate memory	0–15	117	14.3	1.0	15	14–15	11–15
Concentration	0–5	117	3.8	1.2	4	3–5	0–5
Digits backwards	0–4	117	3.0	1.0	3	2–4	0–4
Delayed recall	0–5	117	4.0	1.1	4	3–5	0–5
mBESS balance errors	0–30	117	2.4	2.2	2	1–4	0–9
Double leg stance	0–10	117	0	0	0	0	0–0
Single leg stance	0–10	117	1.8	1.5	2	0–3	0–5
Tandem stance	0–10	117	0.6	1.0	0	0–1	0–5
Tandem gait: Best time (s)	N/A	117	11.9	3.0	12	10–13.5	6.0–25.0
Tandem gait: Mean time (s)	N/A	117	12.4	3.6	12	10–14	6.5–32.25

*SAC, Standardized Assessment of Concussion; mBESS, modified Balance Error Scoring System; IQR, interquartile range; n, sample size. The Tandem Gait test for the SCAT5 is embedded in the “Neurological Screen” and scored as pass or fail. For this baseline testing program, the league retained the SCAT3 version of the Tandem Gait which included 4 timed trials of the test*.

**Table 3:** Cutoff scores and classification ranges for the SCAT5 components for women professional rugby league players.

	**Broadly normal**		**Below/above average**		**Unusually low/high**		**Extremely low/high**	
**Test component**	**Cutoff**	**% in range**	**Cutoff**	**% in range**	**Cutoff**	**% in range**	**Cutoff**	**% in range**
Symptom severity score	0–7	72.6%	8–15	17.1%	16–32	8.5%	≥33	1.7%
Total number of symptoms	0–4	76.1%	5–9	13.7%	10–18	8.5%	≥19	1.7%
SAC total score	≥26	76.9%	24–25	17.9%	22–23	2.6%	≤ 21	2.6%
Orientation	5	80.3%	4	18.8%	N/A	N/A	3	0.9%
Immediate memory	≥14	85.5%	13	8.5%	12	3.4%	≤ 11	2.6%
Concentration	3–5	85.5%	2	8.5%	1	5.1%	0	0.9%
Digits backwards	2–4	88.9%	N/A	N/A	1	10.3%	0	0.9%
Delayed recall	3–5	89.7%	N/A	N/A	2	8.5%	≤ 1	1.7%
mBESS balance errors	0–3	70.9%	4–5	17.9%	6–8	10.3%	≥9	0.9%
Double leg stance	0	100%	N/A	N/A	N/A	N/A	N/A	N/A
Single leg stance	0–3	82.1%	4	12.8%	5	5.1%	≥6	0%
Tandem stance	0–1	80.3%	2	11.1%	3	6.8%	≥4	1.7%
Tandem gait: Best time (s)	≤ 13.5	76.1%	13.6–14.0	15.4%	14.1–20.0	7.7%	≥20.1	0.9%
Tandem gait: Mean time (s)	≤ 14.0	76.9%	14.1–15.3	13.7%	15.4–24.5	7.7%	≥24.6	1.7%

*Broadly normal contains values within the interquartile range; below/above average approximates the cutoff corresponding with the 25/75th percentile ranks; Unusually low/high approximates the cutoff corresponding with the 10/90th percentile ranks; Extremely low/high approximates the cutoff corresponding with the 2/98th percentile ranks. The Tandem Gait test on the SCAT3 was timed and included as a score whereas on the SCAT5 it is embedded in the neurological exam and scored as pass or fail. The National Rugby League Women chose to use the SCAT3 version of the test during their baseline testing program. In the future, these normative data will be expanded and updated to reflect the league transition to the 10-item word list and using an untimed, pass-fail, tandem gait test*.

### Baseline SCAT5 Symptom Reporting

The distributions of the individual symptoms reported on the 22-item symptom scale are presented in Table 4. The median symptom score was 1.0 (M = 3.2, SD = 4.7) and the median symptom severity score was 2.0 (M = 5.4, SD = 8.2). The most commonly endorsed baseline symptoms on the symptom scale were fatigue or low energy (*n* = 39, 33%), trouble sleeping (*n* = 28, 24%), headache (*n* = 27, 23%), neck pain (*n* = 26, 22%), and difficulty remembering (*n* = 25, 21%). In the total sample, 48 of 117 participants (41%) reported no symptoms (Table 5).

**Table 4:** Normative reference values for baseline individual symptom endorsement.

**Variable**	**None**	**Mild**	**Moderate**	**Severe**
	**(0)**	**(1–2)**	**(3–4)**	**(5–6)**
Headache	76.9%	21.4%	1.7%	0%
“Don't feel right”	90.6%	9.4%	0%	0%
“Pressure in head”	86.3%	13.7%	0%	0%
Difficulty concentrating	82.1%	16.2%	0.9%	0.9%
Neck pain	77.8%	14.5%	7.7%	0%
Difficulty remembering	78.6%	16.2%	4.3%	0.9%
Nausea/vomiting	97.4%	2.6%	0%	0%
Fatigue or low energy	66.7%	30.8%	2.5%	0%
Dizziness	90.6%	7.7%	1.7%	0%
Confusion	92.3%	6.8%	0.9%	0%
Blurred vision	86.3%	12.0%	1.7%	0%
Drowsiness	88.0%	10.3%	1.7%	0%
Balance problems	81.2%	17.1%	1.7%	0%
Trouble sleeping	76.1%	13.7%	8.6%	1.7%
Sensitivity to light	88.9%	10.3%	0.9%	0%
More emotional	84.6%	12.0%	2.6%	0.9%
Sensitivity to noise	92.3%	7.7%	0%	0%
Irritability	89.7%	7.7%	2.6%	0%
Feeling slowed down	86.3%	12.8%	0.9%	0%
Sadness	83.8%	12.8%	3.4%	0%
Fogginess	95.7%	4.3%	0%	0%
Nervous/anxious	84.6%	10.3%	4.3%	0.9%

*Values represent the percentage of women endorsing each symptom at the different severity levels. Some rows add up to >100% due to rounding error*.

**Table 5:** Cumulative frequency distribution of the number of symptoms endorsed at baseline.

	**Mild or greater (1–6)**			**Moderate-severe (3–6)**		
**Number of symptoms endorsed**	***f***	***p***	***cp***	***f***	***p***	***cp***
20+	2	1.7%	1.7%	-	-	-
19	0	0.0%	1.7%	-	-	-
18	1	0.9%	2.6%	-	-	-
17	1	0.9%	3.4%	-	-	-
16	1	0.9%	4.3%	-	-	-
15	1	0.9%	5.1%	-	-	-
14	0	0.0%	5.1%	-	-	-
13	2	1.7%	6.8%	-	-	-
12	1	0.9%	7.7%	-	-	-
11	2	1.7%	9.4%	-	-	-
10	1	0.9%	10.3%	-	-	-
9	4	3.4%	13.7%	-	-	-
8	1	0.9%	14.5%	-	-	-
7	3	2.6%	17.1%	2	1.7%	1.7%
6	3	2.6%	19.7%	0	0.0%	1.7%
5	5	4.3%	23.9%	1	0.9%	2.6%
4	9	7.7%	31.6%	0	0.0%	2.6%
3	10	8.5%	40.2%	2	1.7%	4.3%
2	6	5.1%	45.3%	10	8.5%	12.8%
1	16	13.7%	59.0%	17	14.5%	27.4%
0	48	41.0%	100.0%	85	72.6%	100.0%

*f, frequency; p, percentage; and cp, cumulative percentage*.

### SCAT5 Scores and Symptom Reporting

As expected, there was a very high correlation between total symptoms and symptom severity (*r* = 0.99, *p* < 0.001). All other Spearman correlations among the SCAT5 components were non-significant (*p*s > 0.05), with the exception of the relationship between symptom severity and tandem gait: mean time (*r* = 0.40, *p* < 0.001). A clinical reference guide to interpreting SCAT5 performance is presented in Table 6 and sideline SCAT5 results from five women who were medically diagnosed with concussions during the season are presented in Table 7.

**Table 6:** SCAT5 quick reference guide for clinicians (for women who are professional rugby league players).

**Test component**	**Broadly normal**	**Uncommon**
Symptom severity score	0–7	≥16
Total number of symptoms	0–4	≥10
SAC score total	26–30	<24
Orientation	5	3
Immediate memory	14–15	<13
Concentration	3–5	<2
Digits backwards	2–4	<2
Delayed recall	3–5	<2
mBESS balance errors	0–3	≥6
Double leg stance	0	≥1
Single leg stance	0–3	≥5
Tandem stance	0–1	≥3
Tandem gait: Best time (s)	≤ 13.5	≥14.1
Tandem gait: Mean time (s)	≤ 14.0	≥15.4

*Broadly normal scores for each test or measure are provided in the first column. This is the range of scores that most players obtain during baseline preseason testing. Scores in the “uncommon” column occur in ~10% or fewer of women during baseline testing. A woman can have some scores in broadly normal range following a concussion. These are simply normative reference values and they have not been validated as cutoff scores for diagnostic purposes. During a sideline evaluation, the SAC, mBESS, and Tandem Gait normative values are more useful than the normative values for self-reported symptoms. This is because they are performance-based tests whereas symptom reporting is more influenced by the situational context and motivational factors (e.g., the desire to return to play), making it difficult to compare preseason symptom ratings to sideline symptom ratings*.

**Table 7:** Examples of preseason and sideline SCAT5 scores in five women medically diagnosed with concussion.

	**Player #1**		**Player #2**		**Player #3**		**Player #4**		**Player #5**	
**Test component**	**Preseason**	**Sideline**	**Preseason**	**Sideline**	**Preseason**	**Sideline**	**Preseason**	**Sideline**	**Preseason**	**Sideline**
	**Score**	**Score**	**Score**	**Score**	**Score**	**Score**	**Score**	**Score**	**Score**	**Score**
Maddock's questions	N/A	5	N/A	5	N/A	5	N/A	5	N/A	5
Symptom severity score	0	2	4	44^***^	20^**^	1	0	6	6	0
Total number of symptoms	0	1	4	13^**^	16	1	0	6^*^	3	0
SAC total score	27	12^***^	27	26	26	22^**^	29	24^*^	27	28
Orientation	5	2^***^	5	5	5	4^*^	5	3^***^	5	4^*^
Immediate memory	15	7^***^	15	14	15	15	15	14	14	15
Concentration	4	2^*^	5	5	4	3	5	4	4	5
Digits backward	3	1^**^	4	3	3	2	4	3	3	4
Delayed recall	3	1^***^	2^**^	2^**^	2^**^	0	4	3	4	4
mBESS balance errors	1	0	1	5^*^	0	0	5^*^	4^*^	3	3
Double leg stance	0	0	0	0	0	0	0	0	0	0
Single leg stance	1	0	1	3	0	0	5^**^	2	0	2
Tandem stance	0	0	0	2^*^	0	0	0	2^*^	3^**^	1
Tandem gait (Time)	10.8	12	11.4	14^*^	10	14^*^	7	13	19^**^	11

*Based on the normative reference values:*

* *Above Average symptom severity, number of symptoms, and number of errors on the mBESS, and Below Average performance on the SAC;*

** *Unusually High symptom severity, number of symptoms, and number of errors on the mBESS, and Unusually Low performance on the SAC; or*

*** *Extremely High symptom severity, number of symptoms, and number of errors on the mBESS, and Extremely Low performance on the SAC. The five-word memory list was administered; only one trial of the tandem gait was conducted on the sideline for the athletes*.

## Discussion

The current study is the first to provide SCAT5 normative reference values for women who are professional rugby league players. These normative data were collected prior to the beginning of the season, and none of the women were recovering from an injury at the time of data collection. These normative data can improve the clinical interpretation of SCAT5 scores if a player is suspected of having a concussion. The majority of the sample (59%) endorsed at least one baseline symptom, and approximately one in three women (32%) endorsed four or more symptoms at baseline (Table 5). The women in this sample endorsed more preseason symptoms than are endorsed during the preseason by elite male Rugby Union players (Fuller et al., 2018; Tucker et al., 2020b). In our study, the most common symptoms were fatigue or low energy (33%), trouble sleeping (24%), headache (23%), neck pain (22%), and difficulty remembering (21%). These results are consistent with prior literature in that fatigue is typically the most frequent or second most frequent symptom reported during baseline SCAT evaluations across a variety of age groups, geographic regions, and sports (Schneider et al., 2010; Jinguji et al., 2012; Hänninen et al., 2016; Fuller et al., 2018; Petit et al., 2020; Tucker et al., 2020b), and difficulty sleeping is also common (Schneider et al., 2010; Jinguji et al., 2012; Hänninen et al., 2016; Fuller et al., 2018; Tucker et al., 2020b), reflecting that these symptoms occur frequently in daily life (Yengo-Kahn et al., 2016).

Scores on the SAC and mBESS were high but imperfect and were broadly consistent with prior normative studies (Jinguji et al., 2012; Valovich McLeod et al., 2012; Snyder et al., 2014; Hänninen et al., 2016; Snedden et al., 2017; Fuller et al., 2018; Tucker et al., 2020b). Small proportions of the current sample earned perfect scores on SAC delayed recall (8%) and digits backwards (38%), while most of the sample (85%) correctly named the months in reverse. These findings are broadly consistent with a report from male Finnish hockey players, where approximately one quarter of the sample obtained perfect scores on delayed recall and digits backward, and 94% stated the months in reverse without error (Hänninen et al., 2016). The findings are also consistent with a study that included 31 16–19-year-old female athletes from the U.S. Pacific Northwest, where about one quarter of the sample scored perfectly on digits backwards and 84% stated the months of the year in reverse without error (Jinguji et al., 2012).

### Case Examples of Acutely Concussed Athletes

Sideline SCAT5 results from five women who were medically diagnosed with a concussion are presented in Table 7. These cases illustrate three important points. First, some athletes show highly elevated symptom reporting (player #2) or poor performance on cognitive testing (player #1). Second, some athletes endorse very few symptoms and perform well overall on cognitive testing, balance, and tandem gait, making it difficult to determine whether they are experiencing deficits that could be attributable to a concussion. Third, some athletes have baseline preseason test scores that are worse than expected, such as high symptom endorsement (player #3), poor delayed recall on the SAC (player #2 and player #3), poor performance on balance testing (player #4, single leg stance; player #5, double leg stance), or slow tandem gait (player #5). These worse-than-expected scores often are due to situational factors as opposed to representing players' “true” and stable baseline scores.

### Effects of Exercise on SCAT5 Symptom Ratings, Balance, and Cognition

If a player is suspected to have a concussion during a rugby league match, she will be removed from play and medically evaluated. Little is known about the effects of vigorous exercise in general, and match play exertion and adrenaline in particular, on SCAT5 component scores. Two studies have examined exercise in women in a controlled research setting (Gaetz and Iverson, 2009; Chung Pin Yong et al., 2020), and other studies have examined the effects of exercise on SCAT component scores in men (Gaetz and Iverson, 2009; Lee et al., 2017; Tucker et al., 2020a). In these studies, the subjects were healthy at the time of their participation; none had experienced a recent concussion. In a study of collegiate athletes, 45 women completed symptom ratings before and after a vigorous 15-min cycle ergometry protocol (Gaetz and Iverson, 2009). Some women reported pre-post exercise changes in headache (11.1% worsened, 22.2% improved), dizziness (20.0% worsened, 8.9% improved), perceived balance problems (24.4% worsened, 4.4% improved), fatigue (51.0% worsened, 26.6% improved), sensitivity to light (4.4% worsened, 15.5% improved), sensitivity to noise (0% worsened, 8.8% improved), and feeling mentally foggy (13.3% worsened, 15.1% improved) (Gaetz and Iverson, 2009).

During a second study, researchers administered the SCAT3 to 87 women participating in amateur sports, before and after exercise. None of the subjects had experienced a recent concussion. There were no significant pre-post exercise group differences in symptom ratings (total scores), SAC scores, or mBESS errors. However, those women who rated their exertion levels as “hard,” compared to those who rated their exertion as “light,” endorsed more symptoms after exercise (Chung Pin Yong et al., 2020). Interestingly, some women endorsed (as present) a wide range of symptoms following exercise, including headache (12.6%), pressure in head (20.6%), neck pain (19.5%), dizziness (17.2%), perceived balance problems (17.2%), feeling slowed down (40.2%), feeling like in a fog (10.3%), difficulty concentrating (18.4%), and fatigue (62.1%) (Chung Pin Yong et al., 2020).

It is important to note that the women participating in these studies were in a very different context (a research project) compared to if they were assessed during a sporting match—such as the women presented in Table 7 (some of whom reported few or no symptoms, on the sideline, following a blow to the head; Players #1, 3, and 5). This issue of assessment context is discussed, further, in the section below. Still, more research is needed to improve the interpretation of sideline SCAT5 scores in clinical practice.

### Clinical Implications for Using SCAT5 Normative Reference Values

The current study has important clinical implications for the assessment of concussion in women who are professional rugby league players. Regarding the performance-based measures, it is important to appreciate that they lose their sensitivity to the effects of concussion fairly quickly over the first 24–48 h following injury. The cognitive (SAC), balance (mBESS), and coordination (Tandem Gait) tests are fairly crude measures that are useful for measuring acute effects, but, due to spontaneous recovery, athletes tend to perform in the normal range on these tests soon after an injury (McCrea et al., 2013). As seen in Table 7, some women with concussions will perform normally on these tests during their sideline medical evaluation, immediately following their injury. This is why the tests themselves are not “diagnostic” of concussion; they are used as part of a clinical evaluation to measure the possible acute effects of concussion.

There are a number of factors to consider when interpreting the SCAT5 symptom ratings. First, as seen in Tables 4, 5, it is fairly common for these athletes to experience and report some symptoms at baseline, during the preseason. This is because these symptoms are fairly common in daily life. During the preseason, it is common for women to endorse at least one symptom as mild or greater. As seen in Table 5, 59.0% of women endorsed at least one symptom, and nearly one in four women (23.9%) endorsed five or more symptoms at baseline. It is far less common, however, for these women to endorse symptoms as “moderate” or greater in severity. Only 4.3% of the sample endorsed three or more symptoms at this level during preseason testing.

Second, the situational context likely influences symptom reporting. For example, if a player is removed from a professional match and medically evaluated for concussion on the sideline, she is in a dramatically different situational context than when she is filling out this questionnaire during baseline testing. Some symptoms on the scale are irrelevant on the sideline (e.g., trouble sleeping), some are unlikely (e.g., sadness), and most will be interpreted by the player in a different manner than they were interpreted at baseline. That is, instead of thinking about her life in general, she will be assessing her current state and trying to determine if she is experiencing acute concussion symptoms. As seen in Table 7, only one of five acutely concussed players endorsed severe concussion symptoms on the sideline. Therefore, for sideline evaluations, clinicians are encouraged to carefully consider any symptoms that are endorsed and to discuss these symptoms with the athlete.

Third, the results presented in Table 7 were derived from testing that occurred within minutes of injury, and because the neurobiological effects of concussion evolve over time, the scores could worsen over the first few hours following the injury. Fourth, normative data become more relevant for symptom reporting when the athlete is evaluated days or weeks after an injury because then the context has become more consistent with general day-to-day experience of those symptoms. Even then, however, context matters—and athletes assessed in the 1st week following injury are probably conceptualizing their personal experience with the symptoms very differently than when they completed the scale during the preseason. Therefore, the evaluation of subjectively experienced symptoms is complex and nuanced; it requires careful discussion with the athlete to better understand her perspective and frame of reference.

### Limitations

A limitation of the current study is the lack of available demographic information (aside from age), concussion histories, and medical/psychological histories of the participants. This prevented analyses investigating possible associations between these variables and SCAT5 symptom ratings and test performance. None of the athletes, however, were recovering from a concussion at the time of preseason testing. Additionally, the sample size was relatively small (*N* = 117) compared to other normative SCAT research (Schneider et al., 2010; Valovich McLeod et al., 2012; Snyder et al., 2014; Snedden et al., 2017; Fuller et al., 2018), although our sample reflected the entire population of current NRLW players, making external validity excellent in this regard.

## Conclusions

In conclusion, normative SCAT5 reference values for women who are professional rugby league players are provided. The performance-based tests (cognition, balance, and coordination) are useful on the day of injury, but they lose their sensitivity thereafter in most cases. The clinical interpretation of SCAT5 scores following a concussion can be improved by understanding both a person's personal pre-injury baseline performance and normative reference values. The interpretation of SCAT5 results requires clinical expertise and a careful consideration of a broad range of factors that can influence symptom reporting and test performance.

## Data Availability Statement

The datasets presented in this article are not readily available because of privacy considerations. The original contributions presented in the study are included in the article, further inquiries can be directed to the corresponding author. The statistical code, syntax, output, and analyses are available to qualified researchers upon request. Requests should be directed to Andrew Gardner, andrew.gardner@newcastle.edu.au.

## Ethics Statement

The studies involving human participants were reviewed and approved by The University of Newcastle Human ethics Committee. Written informed consent for participation was not required for this study in accordance with the national legislation and the institutional requirements.

## Author Contributions

GI conceptualized the study, assisted with the literature review, helped conceptualize the analyses, and drafted sections of the manuscript. DH helped conceptualized the statistical analyses, conducted the analyses, and drafted the results section. RVP assisted with the statistical analyses, assisted with reviewing the literature, and drafted sections of the manuscript. PB helped oversee data collection. AG served as the principal investigator, secured regulatory ethics for the study, helped conceptualize the study, and collated the data and created the database for analysis. All authors edited the manuscript and approved the final manuscript.

## Conflict of Interest

GI serves as a scientific advisor for NanoDX^®^ (formerly BioDirection, Inc.), Sway Operations, LLC, and Highmark, Inc. He has a clinical and consulting practice in forensic neuropsychology, including expert testimony, involving individuals who have sustained mild TBIs (including athletes). He has received research funding from several test publishing companies, including ImPACT Applications, Inc., CNS Vital Signs, and Psychological Assessment Resources (PAR, Inc.). He has received research funding from the National Football League. He has also received research funding from the Harvard Integrated Program to Protect and Improve the Health of National Football League Players Association Members. PB is the Chief Medical Officer of the National Rugby League. AG serves as a scientific advisor for hitIQ, Ltd. He has a clinical practice in neuropsychology involving individuals who have sustained sport-related concussion (including current and former athletes). He has been a contracted concussion consultant to Rugby Australia since July 2016. He has received travel funding or been reimbursed by professional sporting bodies, and commercial organizations for discussing or presenting sport-related concussion research at meetings, scientific conferences, workshops, and symposiums. Previous grant funding includes the NSW Sporting Injuries Committee, the Brain Foundation (Australia), an Australian-American Fulbright Commission Postdoctoral Award, a Hunter New England Local Health District, Research, Innovation and Partnerships Health Research & Translation Centre and Clinical Research Fellowship Scheme, and the Hunter Medical Research Institute (HMRI), supported by Jennie Thomas, and the HMRI, supported by Anne Greaves. He has also received research funding from the National Rugby League (NRL) to conduct research into the health of retired professional rugby league players. The remaining authors declare that the research was conducted in the absence of any commercial or financial relationships that could be construed as a potential conflict of interest.
